# Risk factors associated with influenza A (H1N1)pdm09: a nested case control study of TB patients with ILI in Lahore District, Pakistan

**DOI:** 10.1186/s12879-024-09263-7

**Published:** 2024-07-26

**Authors:** Gulshan Umbreen, Abdul Rehman, Sadaf Aslam, Chanda Jabeen, Muhammad Iqbal, Aayesha Riaz, Shakera Sadiq, Rubab Maqsood, Hamad Bin Rashid, Saira Afzal, Nimra Arshad, Muhammad Hassan Mushtaq, Mamoona Chaudhry

**Affiliations:** 1 Department of Epidemiology & Public Health, University of Veterinary and Animal Sciences, Lahore, Pakistan; 2 Department of Veterinary Surgery, University of Veterinary and Animal Sciences, Lahore, Pakistan; 3 Department of Patho-Biology, Faculty of Veterinary Animal Sciences, PMAS- Arid Agriculture University, Rawalpindi, Pakistan; 4 Department of Community Medicine, King Edward Medical University, Lahore, Pakistan

**Keywords:** Influenza, Viruses, Risk factors, Tuberculosis (TB)

## Abstract

**Background:**

Co-morbidity with respiratory viruses including influenza A, cause varying degree of morbidity especially in TB patients compared to general population. This study estimates the risk factors associated with influenza A (H1N1)pdm09 in TB patients with ILI.

**Methods:**

A cohort of tuberculosis (TB) patients who were admitted to and enrolled in a TB Directly Observed Therapy Program (DOTs) in tertiary care hospitals of Lahore (Mayo Hospital and Infectious Disease Hospital) were followed for 12 weeks. At the start of study period, to record influenza-like illness (ILI), a symptom card was provided to all the participants. Every participant was contacted once a week, in person. When the symptoms were reported by the participant, a throat swab was taken for the detection of influenza A (H1N1)pdm09. A nested case control study was conducted and TB patients with ILI diagnosed with influenza A (H1N1)pdm09 by conventional RT-PCR were selected as cases, while those who tested negative by conventional RT-PCR were enrolled as controls. All cases and controls in the study were interviewed face-to-face in the local language. Epidemiological data about potential risk factors were collected on a predesigned questionnaire. Logistic analysis was conducted to identify associated risk factors in TB patients with ILI.

**Results:**

From the main cohort of TB patients (*n* = 152) who were followed during the study period, 59 (39%) developed ILI symptoms; of them, 39 tested positive for influenza A (H1N1)pdm09, while 20 were detected negative for influenza A (H1N1)pdm09. In univariable analysis, four factors were identified as risk factors (*p* < 0.05). The final multivariable model identified one risk factor (sharing of towels, *P* = 0.008)) and one protective factor (wearing a face mask, p = < 0.001)) for influenza A (H1N1)pdm09 infection.

**Conclusion:**

The current study identified the risk factors of influenza A (H1N1)pdm09 infection among TB patients with ILI.

**Supplementary Information:**

The online version contains supplementary material available at 10.1186/s12879-024-09263-7.

## Introduction

Influenza and other respiratory viruses pose a threat to the health of the entire world’s population as they are a major cause of disease and mortality each year [1]. The influenza viruses (A and B) are among the major pathogens causing recurrent human epidemics. Influenza A has a greater impact on public health than influenza B due to its rapid evolution and wide range of hosts [2, 3]. According to the data from the Global Burden of Disease study, the mortality rate from lower respiratory tract infections caused by influenza in the WHO Middle East and North Africa (MENA) region reached 0.9 per 100,000 population, with hospitalization rates ranging from 100 to 299 per 100,000 population [4].

Pakistan is the world’s sixth-most populous country, with 40% of its population living in cities with limited healthcare facilities, making it vulnerable to an influenza outbreak [5].

Documented influenza risk factors include (i) individual demographic characteristics such as age (younger people have a higher risk of infection, while older people have a higher risk of complications and mortality), immunodeficiency, pregnancy, respiratory diseases and chronic underlying medical conditions (ii) individual household characteristics such as living with children; and (iii) individual profession such as having contacts with children or infected people [6–8]. Co-morbidity with respiratory viruses including influenza A, cause varying degree of morbidity especially in TB patients compared to general population [9]. In particular, influenza can weaken innate immune responses to secondary bacterial infections by impairing the T cell immunity [10]. Increased mortality has been seen in TB patients following influenza infections [11]. Individuals with pulmonary tuberculosis (PTB) are at a higher risk of being infected with the influenza virus which may lead to chronic lung disease, immunosuppression and even death. A better understanding of the co-infection of influenza and TB is crucial for policymakers to prioritize the target population for influenza vaccination [12].

Thus, interventional prevention against influenza such as vaccination according to the recommendations by World Health Organization (WHO), could help to reduce influenza incidence, hospitalization and case fatality rates [13]. Personal protective measures, on the other hand, such as wearing a face mask, proper hand washing and cleanliness, and some other physical interventions, have shown to be beneficial in preventing disease transmission [14]. To properly target specific interventions, including PPE and vaccination, this study focused on identifying risk factors in TB patients with ILI. As only influenza A (H1N1)pdm09 was detected among patients with ILI, the necessity to determine the specific type of influenza virus associated with these risk factors before implementing interventions becomes crucial.

Our study seeks to bridge a critical gap in existing research by specifically investigating the risk factors associated with influenza among tuberculosis (TB) patients. While numerous studies have extensively explored risk factors for influenza in the general population, there remains an evident absence of comprehensive research focusing on TB patients. The current study aims to provide precise and detailed knowledge designed for TB patients, thereby advancing scientific understanding and informing targeted interventions for this neglected group. Therefore, current study identified the risk factors associated with influenza A (H1N1)pdm09 in TB patients with ILI who were admitted to and enrolled in a TB Directly Observed Therapy Program (DOTs) in tertiary care hospitals of Lahore.

## Methods

### Study design

A nested case-control study was conducted among Influenza A (H1N1)pdm09 positive TB cases with ILI and influenza A negative (H1N1)pdm09 TB controls with ILI.

### Study population

A cohort of TB patients who were admitted to and enrolled in a TB Directly Observed Therapy Program (DOTs) in (Mayo Hospital and Infectious Disease Hospital) were followed for 12 weeks. TB patients who developed ILI and tested positive for influenza A (H1N1)pdm09 by conventional RT-PCR were chosen as cases, while those who tested negative by conventional RT-PCR were chosen as controls. Participants who refused to participate in this study were excluded. Mayo Hospital is one of the oldest and biggest hospitals in Lahore, Punjab. Infectious Disease hospital is specific for infectious diseases in which patients need isolation and specific treatments i.e. Rabies, TB. Mayo Hospital is a general hospital that deals with all kinds of diseases. Mayo and Infectious Disease hospitals were chosen due to their high admission rates for TB patients and accessibility, aiding data collection.

### Study procedure

**Fig. 1:**
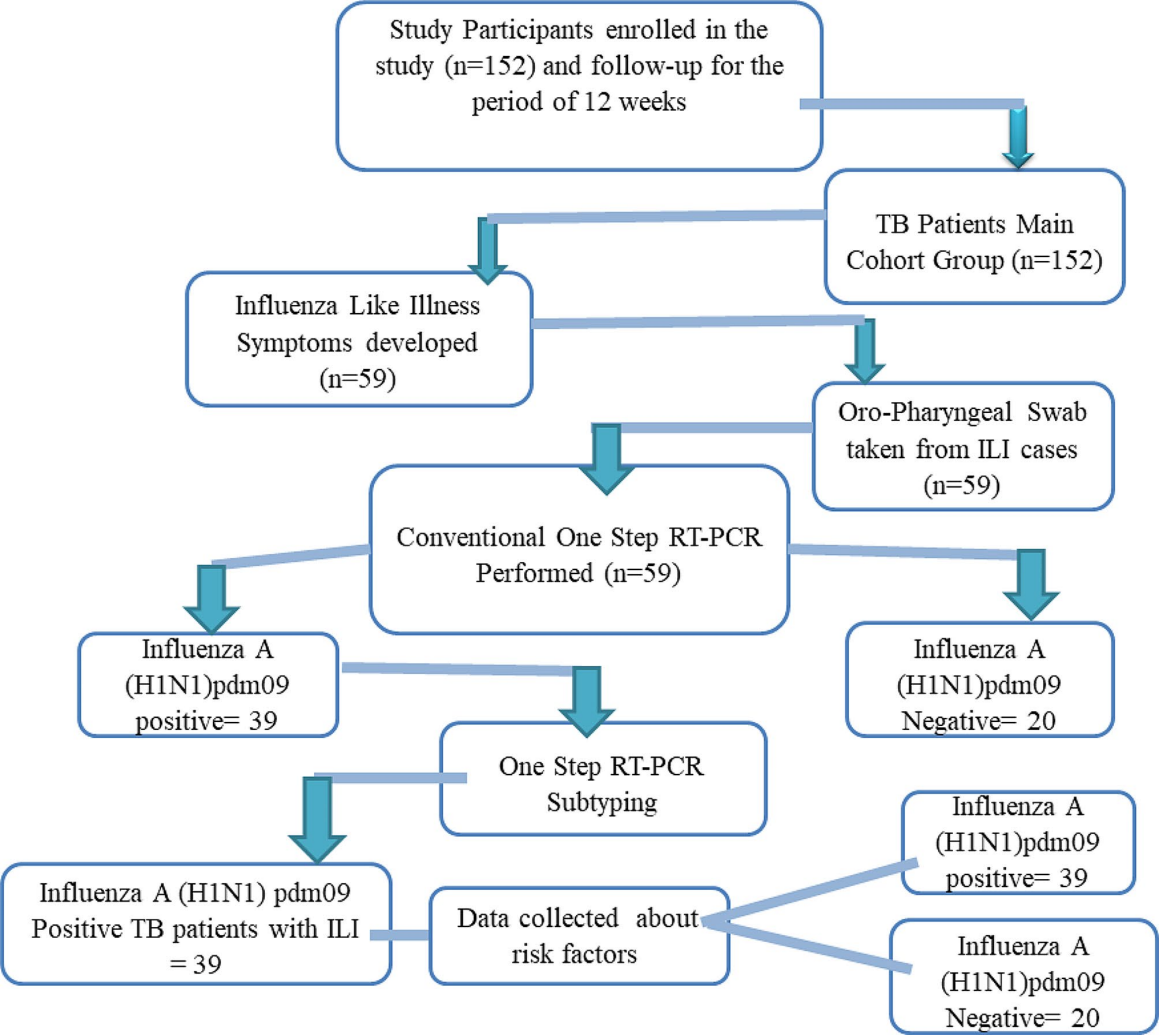
Flow chart of enrolled participants

The data was gathered using a structured questionnaire. All participants who voluntarily agreed to participate in the nested case-control study were given a consent form in local language (Urdu). The sample size was determined based on a previously conducted cohort study, which initially included 152 TB patients as the main cohort [12]. Among these patients, 59 showed ILI symptoms. From those TB patients with ILI, 39 who tested positive for influenza A (H1N1)pdm09 were selected as cases, while the remaining 20 TB patients with ILI, but testing negative for influenza A (H1N1)pdm09, were chosen as controls. All TB patients were followed for a period of 12 weeks regardless of the influenza season. At the start of study period, to record influenza-like illness (ILI), a symptom card was provided to all the participants. Every participant was contacted once a week, in person. When the ILI symptoms were reported, a throat swab was taken for the detection of the influenza A (H1N1)pdm09 (Fig. 1). All cases and controls in the study were interviewed face-to-face in the local language (Urdu). All information about various risk factors (marital status, education, family type, public transportation use, smoking history, respiratory allergy, ventilation system, etc.) was collected on a predesigned questionnaire [15].

### Laboratory procedure

An oropharyngeal swab was collected by a trained healthcare professional registered with the Pakistan Nursing Council within three days of the onset of symptoms. The sample was placed in a cryovial tube with 2–3 mL of viral transport media right away. All specimens were transported at 4 °C to the Disease Surveillance Laboratory, Department of Epidemiology & Public Health, University of Veterinary and Animal Sciences, Lahore, Pakistan. All samples were handled in a biosafety level 2 cabinets (BSL-2). The TRIzol Reagent ® was used to extract viral RNA according to the manufacturer’s instructions [16]. The RNA was either used immediately or stored at -80 °C for future use. Conventional RT-PCR was carried out on a 96-well (Applied Biosystems™ Veriti™ Thermal Cycler) machine using a Qiagen one-step RT-PCR kit following WHO instructions [17]. The reverse transcriptase step was performed at 50 °C for 30 min, 95 °C for 15 min, followed by 35 cycles of amplification (94 °C for 30 s, 55 °C (for M gene)/57°C (for HA gene) for 30 s, 72 °C for 20 s), 72 °C for 7 min, and held at 4 °C using the specific primers [17, 18] (Table 1). To detect the amplified product of RT-PCR, a 2% agarose gel was prepared. A gel documentation system was used to take a digital image of the gel (Supplementary Fig. 1).

**Table 1:** Primers [for M and (H1N1)pdm09] used in the study

Gene	Primers	Sequence
Matrix Gene	Forward Primers	TTCTAACCGAGGTCGAAACG
	Reverse Primers	ACAAAGCG TCTACGCTGCAG
HA Gene	Forward Primers	HKU-SWF:5-GAGCTCAGTGTCATCATTTGAA
	Reverse Primers	HKU-SWR: 5-TGCTGAGCTTTGGG TATGAA-3

### Statistical analysis

The datasets were entered into the EpiData software (version 3.1, Odense, Denmark), verified for errors and inconsistencies by randomly comparing digital data to paper files, and then exported to Excel (version 2013, Microsoft Office, USA) for further processing. For all statistical analyses, R software (version 4.2.1, R Foundation for Statistical Computing, Vienna, Austria) was used. Frequencies and proportions were used to quantify categorical variables. The symptom severity score was calculated using the subjective self-rating of eight symptoms, including a, cough, sore throat, runny nose, fatigue, fever, sneezing, headache, and muscle aches. Each symptom was scored on an ordinal scale, with responses ranging from absent (0) to mild (1), moderate (2), or severe (3) [19]. The association of independent risk factors with the desired outcome i.e. influenza A (H1N1)pdm09 positive and negative in TB patients with ILI was tested using univariable logistic regression analysis. Variables that fulfilled the selection criteria (i.e., *p* ≤ 0.25) were eligible for inclusion in multivariable logistic regression analysis [20]. The odds ratios (ORs) and 95% confidence intervals (CIs) were calculated by using epiR package (version 2-0-60). Model fitness was determined using the likelihood ratio test and Akaike’s information criterion (AIC).

## Results

From the main cohort of TB patients (*n* = 152) who were followed during the study period, 59 (39%) developed ILI symptoms; of them, 39 tested positive for influenza A (H1N1)pdm09, while 20 were detected negative for influenza A (H1N1)pdm09. Among TB patients with ILI, the ratio of males and females was almost equal. The majority was married and had a nuclear family. Details of demographical characteristics are described in (Table 2).

**Table 2:** Socio-demographic characteristics of TB Patients with ILI

Variables		TB Patients with ILI + ve (*n* = 59)	Influenza A (H1N1)pdm09 + ve TB Patients with ILI (39)	Influenza A (H1N1)pdm09 -ve TB Patients with ILI (20)
**Gender**	Male	30 (50.84%)	23 (58.97%)	7 (35%)
	Female	29 (49.15%)	16 (41.02%)	13 (65%)
**Age**	18–30	21 (35.59%)	15 (38.46%)	6 (30%)
	31–43	14 (23.72%)	10 (25.64%)	4 (20%)
	44–55	4 (6.77%)	2 (5.12%)	2 (10%)
	> 55	20 (33.89%)	12 (30.76%)	8 (40%)
**Marital Status**	Un-Married	19 (32.20%)	15 (38.46%)	4 (20%)
	Married	34 (57.62%)	23 (58.97%)	11 (55%)
	Widow	5(8.47%)	1 (2.56%)	4 (20%)
	Divorced	1 (1.69%)	0 (0%)	1 (5%)
**Education**	Illiterate	35 (59.32%)	22 (56.41%)	13 (65%)
	Primary	10 (16.94%)	7 (17.94%)	3 (15%)
	Secondary	13 (22.03%)	9 (23.07%)	4 (20%)
	Intermediate	0 (0%)	0 (0%)	0 (0%)
	Graduation/ Post-Graduation	1(1.69%)	1 (2.56%)	0 (0%)
**Occupation**	Employed	25 (42.37%)	17(43.58%)	8 (40%)
	Un-Employed	28 (47.45%)	19 (48.71%)	9 (45%)
	Housewife	6 (10.16%)	3 (7.69%)	3 (15%)
**Residence**	Rural	12 (20.33%)	6 (15.38%)	6 (30%)
	Urban	47 (79.66%)	33 (84.61%)	14 (70%)
**Monthly Income**	< 15,000	30 (50.84%)	18 (46.15%)	12 (60%)
	15,000–30,000	23 (38.98%)	16 (41.02%)	7 (35%)
	31,000–45,000	4 (6.77%)	3 (7.69%)	1 (5%)
	> 45,000	2 (3.38%)	2 (5.12%)	0 (0%)
**Family Type**	Nuclear Family	39(66.1%)	25 (64.10%)	14 (70%)
	Extended Family	20 (33.8%)	14 (35.89%)	6 (30%)

**Fig. 2:**
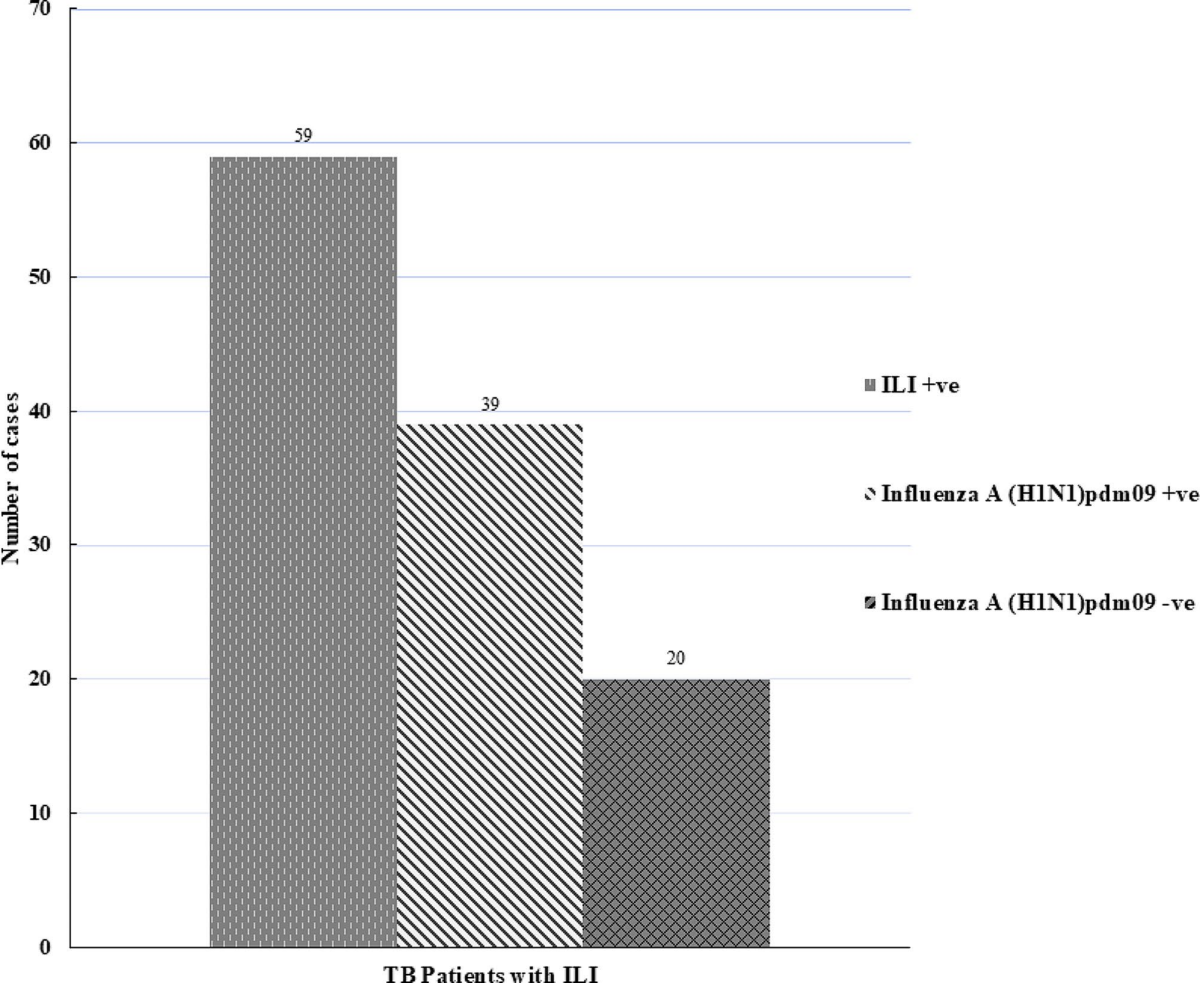
Influenza A (H1N1)pdm09 among 59 TB patients with ILI

ILI was found in 39% (59/152) of the TB main cohort (95% CI: 31.44–46.75) of all enrolled participants (*n* = 152). About 66% (39/59) of the TB patients with ILI (95% CI: 53.37–76.86) tested positive for influenza A (H1N1)pdm09, while 34% (20/59) of the cases tested negative for influenza A (H1N1)pdm09 (Fig. 2).

**Fig. 3:**
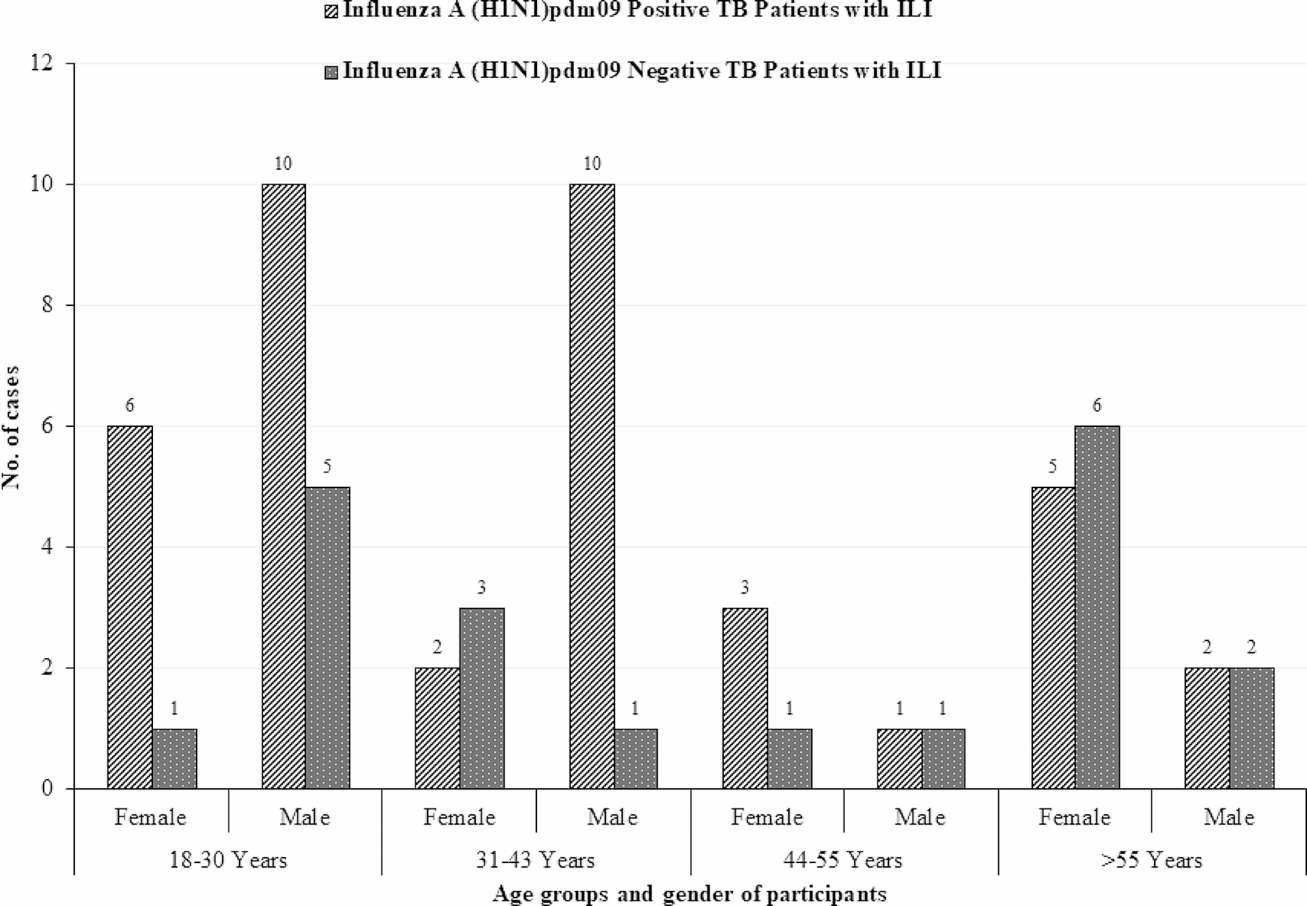
Distribution of Influenza A (H1N1)pdm09 positive and Influenza A (H1N1)pdm09 negative according to age and gender of TB patients with ILI

Male TB patients with ILI were more likely to be infected with influenza A (H1N1), with ages ranging from 18 to 30 years (43.5%, 10/23) and 31 to 43 years (43.5%, 10/23) respectively (Fig. 3).

### Clinical characteristics

**Fig. 4:**
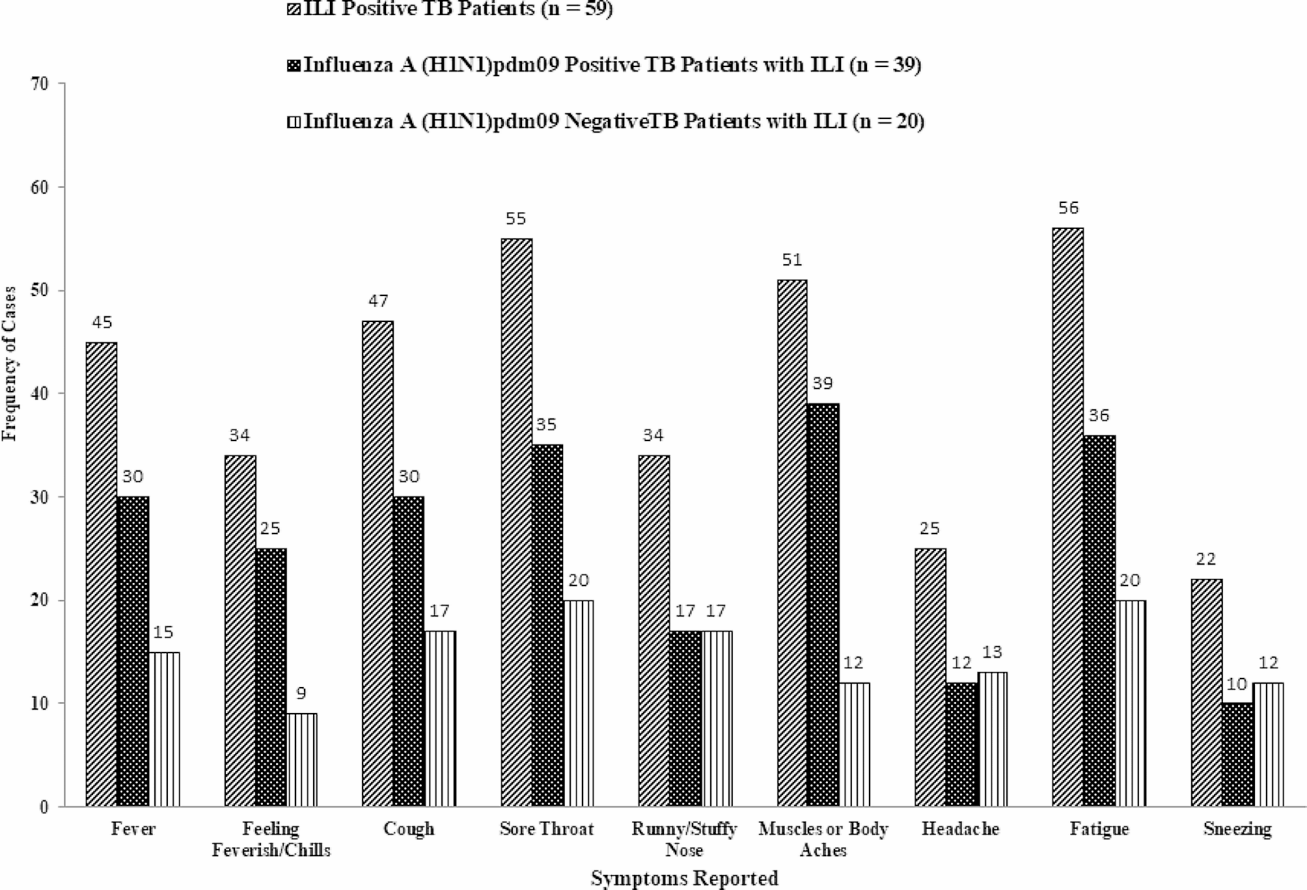
Distribution of clinical symptoms in ILI, Influenza A (H1N1)pdm09 positive cases and Influenza A (H1N1)pdm09 negative control group

Among 59 TB patients with ILI, fatigue (95%) and sore throat (93%) were reported as the most common symptoms.. Muscle aches or body pain (86%), cough (80%), and fever (76%) were the other most common symptoms reported by the ILI group. Feeling feverish (58%), having a runny nose (58%), having a headache (42%), and sneezing (37%), were less frequently reported symptoms by the TB patients with ILI. shown in Fig. 4.

Most of the Influenza A (H1N1)pdm09 negative TB patients with ILI had mild symptoms such as sore throat, coughing, muscle or body aches, fever and fatigue (Table 3).

**Table 3:** Symptoms severity score among TB Patients with ILI

Influenza A (H1N1)pdm09 + ve TB Patients with ILI (*n* = 39)					Influenza A (H1N1)pdm09–ve TB Patients with ILI (*n* = 20)			
Symptoms	No Symptoms (0)	Mild Symptoms (1)	Moderate Symptoms (2)	Severe Symptoms (3)	No Symptoms (0)	Mild Symptoms (1)	Moderate Symptoms (2)	Severe Symptoms (3)
Sore throat	4(10.25%)	23(28.97%)	7(17.94%)	5(12.82%)	2 (10%)	9 (45%)	4 (20%)	5 (25%)
Cough	9(23.07%)	21(53.84%)	6(15.38%)	3(7.69%)	5 (25%)	8 (40%)	5 (25%)	(%)2(10%)
Fever	9(23.07%)	24(61.53%)	4(10.25%)	2(5.12%)	3 (15%)	7 (35%)	7 (35%)	3 (15%)
Fatigue	3(7.69%)	20(51.28%)	7(17.94%)	9(23.07%)	1 (5%)	12 (60%)	4 (20%)	3 (15%)
Muscle or body aches	0(0%)	23(58.97%)	11(28.20%)	5(12.82%)	5 (25%)	8 (40%)	6 (30%)	1 (5%)
Running Nose	22(56.41%)	9(23.07%)	4(10.25%)	4(10.25%)	9(45%)	5 (25%)	4 (20%)	2 (10%)
Feverish/chills	14(35.89%)	18(46.15%)	4(10.25%)	3(7.69%)	10 (50%)	4 (20%)	3 (15%)	3 (15%)
Headache	25(64.10%)	11(28.20%)	3(7.69%)	0(0%)	8(35%)	9 (45%)	2 (10%)	1(5%)
Sneezing	29(74.35%)	7(17.94%)	2(5.12%)	1(2.56%)	4 (20%)	11 (55%)	2 (10%)	3 (15%)

The univariable analysis demonstrated three independent risk factors and one protective factor for influenza A (H1N1)pdm09 with *p* value ≤ 0.25 (selection criteria for building a multivariable model). The use of public transport (*p* = 0.002), sharing towels (*p* = 0.02), and a history of smoking (*p* = 0.002) showed strong positive associations with influenza A (H1N1)pdm09 infection. Wearing a face mask *(p = <* 0.001) was identified as a protective factors that showed a strong positive association with influenza A (H1N1)pdm09 infection prevention. (Table 4).

**Table 4:** Univariable analysis of potential risk factors associated with Influenza A (H1N1)pdm09 with p value ≤ 0.25 among TB patients with ILI

Risk/Protective Factors	Response Level	IAV Positive Cases	IAV Negative Control	OR	95%CI	p value
Sharing of Towels	Yes	22	5	3.88	1.18–12.81	0.02^*^
	No	17	15			
Use of Public Transport	Yes	38	14	16.29	1.8 -147.55	0.002^**^
	No	1	6			
History of Smoking	Yes	19	2	8.55	1.74–41.92	0.002**
	No	20	18			
Wearing a Face Mask	Yes	30	4	0.08	0.02–0.28	< 0.001^**^
	No	9	16			

The final model detected one risk factor (sharing of towels, *p* = 0.008) and one protective factor (wearing a face mask, *p* = < 0.001) for Influenza A (H1N1)pdm09, that was best fitted to the model (Table 5).

**Table 5:** Multivariable analysis of potential risk factors associated with Influenza A (H1N1)pdm09 with p value ≤  0.05 among TB patients with ILI

Risk/Protective Factors	Response Level	IAV Positive Cases	IAV Negative Control	OR	95%CI	p value
Sharing of Towels	Yes	22	5	3.88	1.45–32.5	0.008^**^
	No	17	15			
Wearing a Face Mask	Yes	30	4	0.08	0.01–0.28	< 0.001^**^
	No	9	16			

## Discussion

Individuals with pulmonary tuberculosis (PTB) are at higher risk for severe influenza virus infections and co-morbidity with respiratory viruses including influenza A, cause varying degree of morbidity especially in TB patients compared to general population [9, 21]. The identification and reduction of risk factors is most important in primary interventions for health promotion and maintenance [22]. To properly target specific interventions, including PPE and vaccination, the study focused on identifying risk factors associated with influenza positivity in TB patients with ILI. As only influenza A (H1N1)pdm09 was detected among TB patients with ILI, the necessity to determine the specific type of influenza virus associated with these risk factors before implementing interventions becomes crucial. Therefore, this study estimates the risk factors associated with influenza A (H1N1)pdm09 infection in tuberculosis patients. Univariable analysis indicated that individuals who used public transport had a 16 times higher risk of getting influenza infection compared to those who used their own transport. There is a possibility that transmission occurred among passengers due to their close proximity with an infected individual. The duration and frequency of travel may have increased the chances of flu transmission [23, 24]. Various earlier studies have demonstrated that using public transport increases the risk of viral transmission due to the correlation between recent use of public transport and the onset of influenza symptoms [15, 25]. Smoking was also found to be a risk factor in the current study. The results showed that smokers were 8.55 times more likely to be infected as compared to non-smokers. Previously, it was estimated that smokers werefive times more likely than non-smokers to develop laboratory-confirmed influenza [26]. Smoking increases the risk of respiratory diseases [27, 28]. It is also a well-identified risk factor for the development and progression of infectious diseases like influenza viruses [26, 29]. Smoking has been demonstrated to inhibit the response of lung T cells to influenza viruses, which increases susceptibility to infection [30]. Smoking increases the risk of infection and worsening of the disease (including increased mortality and duration of the disease), which is caused by exacerbating innate and adaptive inflammatory responses [28]. Another significant factor reported in current study was towel sharing (OR = 3.88), which was linked to the development of influenza. When towels and handkerchiefs are used to dry or wipe the face and hands, viruses can be transmitted from contaminated surfaces, increasing the risk of influenza and other respiratory infectious diseases. According to previous studies, sharing towels is a risk factor for outbreaks of respiratory infectious diseases [31, 32].

In this study, wearing a face mask was found to be a significant protective factor against transmission of influenza. The current findings have been supported by various earlier studies [33–35]. The use of masks not only protects healthy people, but also decreases the transmission during infectiousness period of asymptomatic and symptomatic carriers, reduction in the number and efficacy of transmission sources within the population. Second, it is expected that wearing a mask will influence behavior. Wearing a mask can increase awareness of the risk of infection as well as the importance of further preventive behaviors such as frequent hand washing, avoiding physical contact, and avoiding crowded public places. Finally, wearing a mask is the most effective way to prevent airborne transmission, which can result in most serious cases of influenza [36]. In this study, fatigue, cough, muscle aches and fever were the most common reported symptoms. Similarly, these symptoms have also reported in earlier studies of laboratory confirmed influenza cases [19, 20]. This study focused solely on influenza A (H1N1)pdm09 risk factors and did not further study any other respiratory pathogens. The identification of risk factors will guide to design interventions to reduce the burden of influenza co-infection among TB patients. Our study findings are also valuable for healthcare officials and policymakers/decision makers who are involved in influenza prevention and control programs. This study was part of a previous cohort study; thus, the sample size was not large enough, and the presence of a zero-cell value in 2 × 2 contingency tables, as a result, many factors were not studied.

## Conclusion

The current study identified the risk factors of influenza A (H1N1)pdm09 infection among tuberculosis patients. Behavioral factors are associated with influenza transmission as well play a role in influenza prevention. Human behavior may contribute to the breakdown of the chain of infection. Modification of behavioral factors could lower the risk of influenza A (H1N1)pdm09 infection and can prevent the outbreaks among TB patients. Further study should be conducted for the more comprehensive picture.

### Electronic supplementary material

Below is the link to the electronic supplementary material.

Supplementary Material 1

## Data Availability

All data and material involved in the current study are available from the corresponding author on reasonable request.
