# Supplementary material for: Risk factors associated with influenza A (H1N1)pdm09: a nested case control study of TB patients with ILI in Lahore District, Pakistan
Source: BMC Infect Dis. 2024 Jul 26;24:741. doi: 10.1186/s12879-024-09263-7 (PMC11282588; doi:10.1186/s12879-024-09263-7)
Supplement: Supplementary file 1 — Supplementary Material 1 [file 12879_2024_9263_MOESM1_ESM.docx]

Detection of influenza A (HINI)pdm09 virus by RT-PCR.


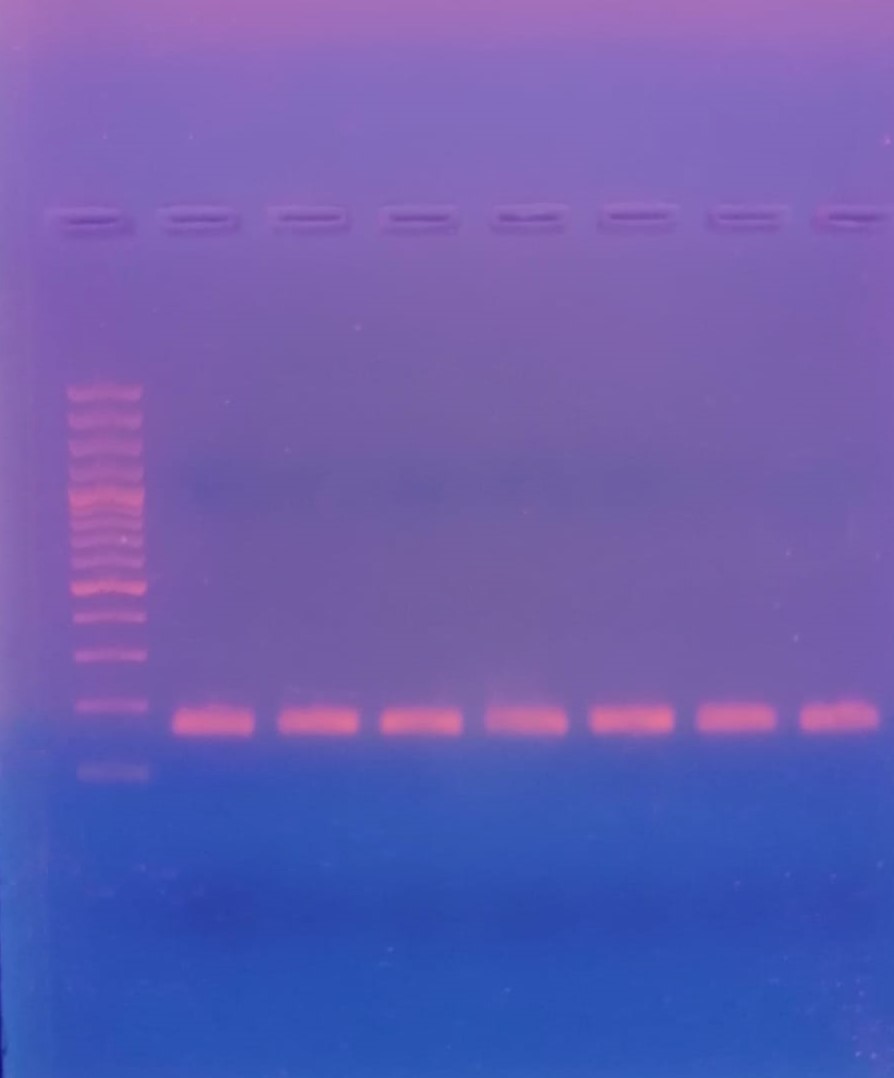


**Laboratory Analysis:**

**Supplementary Fig 1:** Detection of influenza A (HINI)pdm09 at 173bp among TB Patients with ILI: Lane: Ladder 100bp, 1-6 samples and 7 positive control at 2% agrose gel electrophoresis
